# Ergonomic risk profile of critically ill patients

**DOI:** 10.1186/2197-425X-3-S1-A560

**Published:** 2015-10-01

**Authors:** JA Souza Junior, J Silva, M Cosme, L Ayako, C Ayres, K Zarco, L Candido, C Nascimento, FFP Rodrigues, CL Batista, EL Brasil, L Souza, D Carnieli-Cazati, RC Eid, KT Timenetsky

**Affiliations:** Pacientes Graves, Hospital Albert Einstein, São Paulo, Brazil

## Introduction

In early mobilization context as also for any type of critically ill patient mobility, there are different levels of patients' participation during the activity, going from total dependence to independence. In this sense, two aspects should be considered as the efficacy of safe procedures to the patient as well as for the health care team in order to minimize ergonomic risks. This ergonomic risk can be tracked through a specific scale, although so far there are no evidence of this ergonomic risk in critically ill patients.

## Objective

To evaluate the ergonomic risk of critically ill patients.

## Methods

A prospective cohort study was performed from December 2014 through March 2015. A specific and validated ergonomic risk scale that evaluates patients' weight, height, level of consciousness, bed mobility, walking, number of catheters and equipment, as for the patient ambient was applied at the first physiotherapy session and after critically ill department discharge. This scale classifies the patient as high, medium, or low ergonomic risk.

## Results

During the study period 250 patients were evaluated, though 25 were excluded due to incomplete data. From the 225 patients, at the first evaluation, 20% had high ergonomic risk, 63% had medium risk and 18% low risk. At critically ill department discharge there was 15% of high ergonomic risk patients, 56% medium risk and 28% low risk patients. At critically ill department discharge, 5% of patients worsen their ergonomic risk, 19% improved, and 76% remained unchanged.

## Conclusion

In this study most critically ill patients had medium ergonomic risk, and this risk maintained unchanged through patients' hospital stay. This data suggests a higher patient demand at our critically ill department, representing the need for human resources planning to attend this patient condition. It also alerts the health care team for ergonomic risk while caring for this complex patient profile, in order to improve the resources needed to minimize risks for the patient as for the health care team.

## Grant Acknowledgment

This research received no grant from any funding agency in the public, commercial, or not-for-profit sectors.
